# Mapping and characterization of the amplicon near *APOA2 *in 1q23 in human sarcomas by FISH and array CGH

**DOI:** 10.1186/1476-4598-4-39

**Published:** 2005-11-07

**Authors:** Stine H Kresse, Jeanne-Marie Berner, Leonardo A Meza-Zepeda, Simon G Gregory, Wen-Lin Kuo, Joe W Gray, Anne Forus, Ola Myklebost

**Affiliations:** 1 Department of Tumour Biology, The Norwegian Radium Hospital, Oslo, Norway; 2 Faculty of Medicine, University of Oslo, Norway; 3 Center for Human Genetics, Duke University Medical Center, Durham, USA; 4 Comprehensive Cancer Centre, University of California San Francisco, USA; 5 Department of Molecular Biosciences, University of Oslo, Norway

## Abstract

**Background:**

Amplification of the q21-q23 region on chromosome 1 is frequently found in sarcomas and a variety of other solid tumours. Previous analyses of sarcomas have indicated the presence of at least two separate amplicons within this region, one located in 1q21 and one located near the apolipoprotein A-II (*APOA2*) gene in 1q23. In this study we have mapped and characterized the amplicon in 1q23 in more detail.

**Results:**

We have used fluorescence *in situ *hybridisation (FISH) and microarray-based comparative genomic hybridisation (array CGH) to map and define the borders of the amplicon in 10 sarcomas. A subregion of approximately 800 kb was identified as the core of the amplicon. The amplification patterns of nine possible candidate target genes located to this subregion were determined by Southern blot analysis. The genes activating transcription factor 6 (*ATF6*) and dual specificity phosphatase 12 (*DUSP12*) showed the highest level of amplification, and they were also shown to be over-expressed by quantitative real-time reverse transcription PCR (RT-PCR). In general, the level of expression reflected the level of amplification in the different tumours. *DUSP12 *was expressed significantly higher than *ATF6 *in a subset of the tumours. In addition, two genes known to be transcriptionally activated by *ATF6*, glucose-regulated protein 78 kDa and -94 kDa (*GRP78 *and *GRP94*), were shown to be over-expressed in the tumours that showed over-expression of *ATF6*.

**Conclusion:**

*ATF6 *and *DUSP12 *seem to be the most likely candidate target genes for the 1q23 amplification in sarcomas. Both genes have possible roles in promoting cell growth, which makes them interesting candidate targets.

## Background

Gains or amplification of the long arm of chromosome 1 is among the most common chromosomal abnormalities in human neoplasia [1]. Local gains or high-level amplification affecting 1q21-q23 is particularly frequent, and was first described for sarcomas [2-4]. Sarcomas are a heterogeneous group of malignant tumours of various supporting- and connective tissues, ranging from the almost benign well-differentiated liposarcomas (WDLS) to aggressive tumour forms, such as fibrosarcomas, osteosarcomas and malignant fibrous histiocytomas (MFH) [5]. Although infrequent, these tumours have been widely studied at the molecular level, and this has provided insight into mechanisms of importance for tumour development in general. Notably, amplification and over-expression of *MDM2 *and *CDK4*, representing alternative pathways for inactivation of the tumour suppressors p53 and pRb, respectively, were first described for this group of tumours [6,7].

A variety of solid tumours show amplification of 1q21-q22, for instance 70–80 % of hepatocellular carcinomas and 25–30 % of ovarian cancers [8,9]. Studies of renal clear cell, hepatocellular and colorectal carcinomas revealed a higher frequency of 1q21-q23 gains in metastatic tumours [10,11], and gains of 1q21-q25 showed a trend toward short overall survival in high-grade osteosarcomas and neuroblastomas [12,13]. However, also sarcomas of borderline malignancy, such as WDLS, show frequent amplification of 1q21-q23 [2,3,14], and 1q21 is recurrently gained in desmoid tumours [15].

A more detailed molecular analysis of sarcomas has indicated the presence of at least two separate amplicons within 1q21-q23, located in 1q21 and 1q23, respectively [16]. The amplicon in 1q21 was most frequent, and was represented by a yeast artificial chromosome (YAC) clone, 789f2, located proximal to the S100-genes. The YAC detected high amplification levels, and was subsequently used to clone three novel candidate target genes that are highly amplified and over-expressed in both sarcomas and breast cancer [17]. The 1q23 amplicon was first observed in one single tumour (LS21), where a probe for the apolipoprotein A-II (*APOA2*) gene detected high amplification levels [16]. Furthermore, the extent of the amplified region around 1q21 as observed in comparative genomic hybridisation (CGH) analyses was variable, covering only 1q21 in some tumours and 1q21-q23 or -q25 in other tumours [3,4]. These observations indicate that also amplified genes located more distally of 1q21 may be of importance in sarcoma development or progression.

We have now used a bacterial artificial chromosome (BAC) contig of approximately 7 Mb, according to Ensembl (, assembly December 14, 2004) [18] and the UCSC Genome Browser (, assembly May 2004) [19], to map and characterize the amplified region around *APOA2 *in LS21 using fluorescence *in situ *hybridisation (FISH). The two BACs that showed particularly high copy numbers were further analysed in nine additional sarcomas known to have gains of the 1q21-q23 region [3,16] (and unpublished results). Amplicon mapping was also done by microarray-based CGH (array CGH). The amplification patterns of nine possible candidate target genes were determined by Southern blot analysis, and quantitative real-time reverse transcription PCR (RT-PCR) was used to determine the expression levels of the two candidate target genes that showed the highest level of amplification. In addition, the expression level of two genes transcriptionally activated by one of the candidate target gene was analysed.

## Results

### Mapping of the 1q23 amplicon by FISH

**Figure 1:**
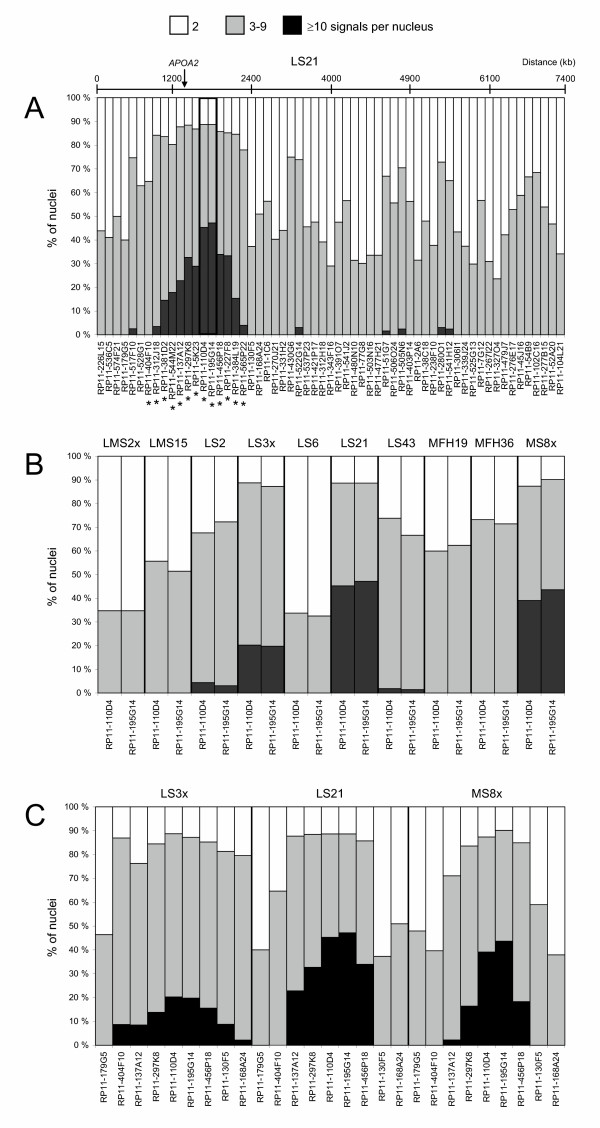
**DNA copy number by FISH**. **(A) **FISH analysis with 59 BACs on interphase nuclei from liposarcoma LS21. The BACs are listed from the centromeric to the telomeric side. The colour of the shading indicates the range of signals observed per nucleus, and the area the percentage of nuclei within each group of signals. The localisation of *APOA2 *in BAC RP11-297K8 is indicated, as well as the distance position of some of the BACs (the intervals are not equal since the BACs are not distributed evenly throughout the region). The two BACs used further in Figure 1B are highlighted, and the BACs used for array CGH in Figure 2 are marked with an asterisk (*). There is a gap of approximately 200 kb between BACs RP11-297K8 and RP11-5K23. After these analyses were performed, the gap was covered by the BAC RP11-122G18. Since no more material is available from LS21, this BAC has not been tested. **(B) **FISH analysis with BACs RP11-110D4 and RP11-195G14 on interphase nuclei from 10 sarcomas (LMS, leiomyosarcoma; LS, liposarcoma; MFH, malignant fibrous histiocytoma; MS, malignant schwannoma, suffix 'x', xenograft). **(C) **FISH analysis with four BACs proximal and three distal of RP11-110D4 and RP11-195G14 on interphase nuclei from LS3x, LS21 and MS8x.

Fifty-nine overlapping BACs, covering approximately 7 Mb in the q23 region of chromosome 1, were hybridised to interphase nuclei from liposarcoma LS21. Figure 1A shows the copy numbers detected by all the BACs.

Copy numbers varied throughout the region, but a subregion defined by 12 overlapping BACs, RP11-312J18 through RP11-565P22, was highly amplified in this tumour compared to the flanking areas. Furthermore, two BACs within this amplified unit, RP11-110D4 and RP11-195G14, detected higher copy numbers than any of the other BACs tested. These two BACs are located approximately 400 kb distal to the *APOA2 *gene that originally identified this amplicon. *APOA2 *is located in BAC RP11-297K8 according to Ensembl. The localisation of BACs RP11-110D4 and RP11-195G14 to 1q23 was confirmed by hybridisation to metaphase nuclei from normal leukocytes (not shown).

To further analyse the amplification frequency of the sequences represented by these two BACs, we determined the copy numbers in nine additional sarcomas known to have gains of 1q21-q23 [3,16], and unpublished results]. The results are presented in Figure 1B. In all cases, amplification was detected in a substantial fraction of the nuclei, especially for LS3x and MS8x. Only two tumours, LMS2x and LS6, showed normal copy numbers in more than 60 % of the nuclei.

The borders of the amplicon were in addition defined for LS3x and MS8x, where high-level amplification was found in 20–50 % of the nuclei. Four BACs located proximal and three located distal to the two BACs detecting the highest copy number were hybridised to interphase nuclei (Figure 1C). The amplicon in LS3x was represented by a few more BACs than the amplicon in LS21 and MS8x. For all three tumours, BACs RP11-110D4 and RP11-195G14 detected the highest level of amplification.

### Mapping of the 1q23 amplicon by array CGH

Mapping of the 1q23 amplicon was also done by array CGH. The 10 tumours analysed by FISH were hybridised to a genomic microarray covering the minimal tiling-path of the 1q12-q25 region. The results from 15 BACs, covering the region in 1q23 that showed highest amplification level by FISH (Figure 1A), are presented here. The dataset with these 15 BACs can be viewed in the microarray database ArrayExpress (, accession number E-MEXP-427) [20].

**Figure 2:**
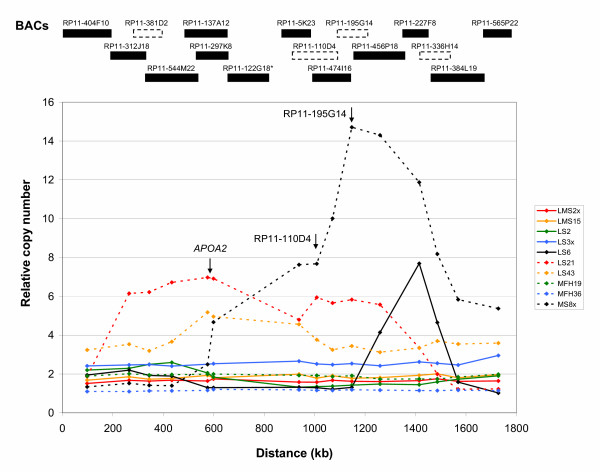
**DNA copy number by array CGH**. Relative DNA copy number of the region represented by BACs RP11-404F10 through RP11-565P22 in 10 sarcomas. The localisation of BACs RP11-381D2, RP11-110D4, RP11-195G14 and RP11-336H14 is based on previous contig maps from the Wellcome Trust Sanger Institute (dotted line), since these are removed from the minimal tiling-path in Ensembl. *BAC RP11-122G18 is not present in the array.

Figure 2 shows the relative copy numbers compared to a normal reference of the overlapping BACs. In four of the tumours, amplification levels above 3-fold were observed, whereas the relative copy number of all the BACs was lower in the other six tumours. For MS8x, the subregion covered by BACs RP11-5K23 through RP11-384L19 showed the highest copy number, with RP11-195G14 and RP11-456P18 detecting relative copy numbers of more than 14. For LS6, the highest copy number was detected by RP11-227F8. LS21 showed relative copy numbers of 5–6 of the region covered by RP11-5K23 through RP11-456P18, but a higher level of the region covered by BACs RP11-312J18 through RP11-297K8, with RP11-137A12 and RP11-297K8 detecting a relative copy number of 7. These two BACs detected the highest copy number also in LS43.

The array CGH results showed in addition other clusters of BACs detecting high copy numbers (data not shown), indicating the presence of several separate amplicons within the 1q12-q25 region, which will be described in a later publication.

### Identification of candidate target genes

Ensembl and the UCSC Genome Browser were used to search for possible candidate target genes. Based on sequence information, these programs visually display possible open reading frames, as well as predicted and known genes.

BACs RP11-110D4 and RP11-195G14, used here for FISH and array CGH, have later been omitted from the minimal tiling-path (Ensembl). The overlapping BACs RP11-5K23, RP11-474I16 and RP11-456P18, which constitute the minimal tiling-path, now represent the corresponding region.

Within the approximately 800 kb region covered by the BACs RP11-5K23 through RP11-384L19, 11 genes were mapped according to Ensembl. The genes are *FCGR2A*, -*2B*, and -*3A*, a family of low affinity immunoglobulin gamma FC receptors; *HSPA6*, heat shock 70 kDa protein 6; *DUSP12*, dual specificity phosphatase 12; *ATF6*, activating transcription factor 6; *OLFML2B*, olfactomedin-like 2B; three genes referred to by RefSeqID: NP_116127, FC receptor homolog expressed in B cells (*FREB*); NP_055512, C-terminal PDZ domain ligand of neuronal nitric oxide synthase (*CAPON*) and NP_001002901, with no description; and one "novel" gene.

The UCSC Genome Browser indicated the same genes as Ensembl, and in addition *FCGR3B*, another member of the low affinity immunoglobulin gamma FC receptors family.

### Southern blot analysis of the candidate target genes

**Figure 3:**
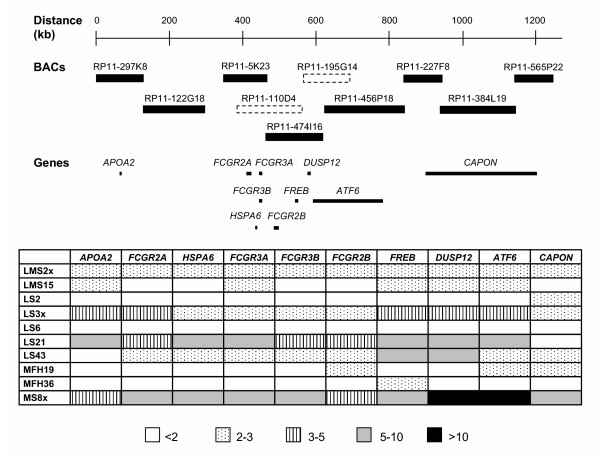
**Gene copy number by Southern blot analysis**. Relative copy number of nine of the candidate target genes normalized with *APOB *in 10 sarcomas. The localisation of BACs RP11-110D4 and RP11-195G14 is based on previous contig maps from the Wellcome Trust Sanger Institute (dotted line), since these are removed from the minimal tiling-path in Ensembl. Gene localisations are based on Ensembl and the UCSC Genome Browser, with the genes listed from the centromeric to the telomeric side. Of the genes located in BACs RP11-297K8 and RP11-122G18, only *APOA2 *is shown. The densitometrically determined levels of amplification are divided into five categories as indicated.

Copy numbers of nine of the candidate target genes were further analysed by Southern blot analysis. The relative copy numbers of the genes compared to *APOB *are presented in Figure 3. The candidate target genes that were not tested were one "novel" gene, one gene with RefSeqID (both with no description), and *OLFML2B*, which appeared in Ensembl after the analyses were completed.

For tumours LS3x, LS21, LS43 and MS8x, the genes *ATF6 *and *DUSP12 *showed the highest amplification level in general, 3–5 fold in LS3x and 5–10 fold in LS21 and LS43 (except *ATF6*), and particularly high copy numbers (>10 fold) in MS8x. *FREB *also showed high amplification levels, except in MS8x where the level was 5-fold (approximately three times less than *ATF6 *and *DUSP12*). For the other six tumours, the amplification level of all the genes was either normal or 2–3 fold.

*APOA2 *showed the same amplification levels as identified previously [16], except that the copy number increase was determined to be 5–10 fold instead of >10 fold in LS21, and 2–3 fold instead of <2 fold in LMS15, either because of experimental variation or because different parts of the tumours were analysed. *APOA2 *is located in BAC RP11-297K8 (Ensembl). By FISH analysis, RP11-297K8 detected high-level amplification in LS21 (32 % of the nuclei), LS3x (13 % of the nuclei) and MS8x (16 % of the nuclei) (Figure 1C), as one would expect.

### Expression analysis of the candidate target genes by quantitative real-time RT-PCR

**Figure 4:**
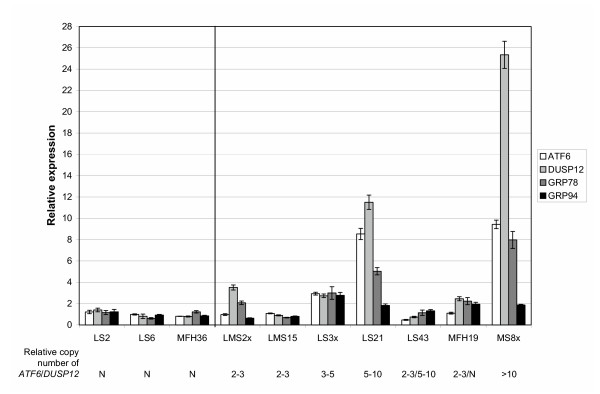
**Gene expression level by quantitative real-time RT-PCR**. Relative expression level of the genes *ATF6, DUSP12, GRP78 *and *GRP94 *in 10 sarcomas. The expression levels have been normalized with the average expression of the endogenous control genes *B2M, GAPDH *and *TBP*. The average relative expression level of the tumours with normal copy number of *ATF6 *and *DUSP12 *(LS2, LS6 and MFH36) has been set to 1. The relative copy number of *ATF6 *and *DUSP12 *determined by Southern blot analysis (Figure 3) is also shown.

Quantitative real-time RT-PCR was used to determine the expression levels of the two genes *ATF6 *and *DUSP12*, which showed the highest level of amplification by Southern blot analysis. In addition, the expression level of two genes known to be transcriptionally activated by *ATF6 *was analysed, *GRP78 *and *GRP94*, glucose-regulated protein 78 kDa and -94 kDa. Figure 4 shows the expression level of *ATF6, DUSP12, GRP78 *and *GRP94 *compared to the average expression of the endogenous control genes β-2-microglobulin (*B2M*), glyceraldehyde-3-phosphate dehydrogenase (*GAPDH*) and TATA box binding protein (*TBP*). The average relative expression level of the tumours with normal copy number of *ATF6 *and *DUSP12 *(LS2, LS6 and MFH36) has been set to 1.

LS21 and MS8x showed the highest level of expression of both candidate target genes, *ATF6 *was over-expressed 8–9 fold whereas *DUSP12 *was over-expressed 11-fold in LS21 and 25-fold in MS8x. LS3x showed 3-fold over-expression of both genes, and for LMS2x and MFH19, *DUSP12 *was over-expressed 2–3 fold whereas *ATF6 *was expressed at the same level as the three tumours with normal copy number. LMS15 and LS43 showed the same expression levels of both *ATF6 *and *DUSP12 *as the tumours with normal copy number of the genes.

*DUSP12 *was significantly higher expressed than *ATF6 *in LMS2x, MFH19, MS8x (all p < 0,001), LS21 and LS43 (both p < 0,01), whereas *ATF6 *was significantly higher expressed than *DUSP12 *in LMS15 (p < 0,01). There was no significant difference in expression of the two genes in the rest of the tumours.

*GRP78 *and *GRP94 *were over-expressed in the same tumours that showed over-expression of *ATF6*. *GRP78 *was over-expressed 8-fold in MS8x, 5-fold in LS21 and 3-fold in LS3x, whereas *GRP94 *was over-expressed 3-fold in LS3x and 2-fold in LS21 and MS8x. In addition, MFH19 showed a 2-fold increase of both genes, whereas LMS2x showed a 2-fold increase for only *GRP78*. LMS15 and LS43 showed the same expression level of both *GRP78 *and *GRP94 *as the tumours with normal copy number of *ATF6 *and *DUSP12*, as they did for *ATF6 *and *DUSP12*.

## Discussion

CGH analyses of sarcomas show that the amplified part of 1q is variable. In some tumours, only 1q21 is highly amplified, whereas other cases have amplification of the whole 1q21-q23 or 1q21-q25 region [3,4]. These observations suggest the presence of multiple, separate amplicons each containing target genes expected to be important for the development or progression of these tumours.

In this study, we did FISH with a contig of BACs spanning 7 Mb around *APOA2 *to map and characterize this amplicon in more detail in liposarcoma LS21, which showed the highest copy number of *APOA2 *in previous analyses [16]. Our results showed that the core of the amplicon could be defined by 12 partly overlapping BACs (Figure 1A). The two BAC clones that detected the highest amplification levels, RP11-110D4 and RP11-195G14, were located approximately 400 kb distally of *APOA2*. Partial mapping of the amplicon in two other tumours confirmed that the most amplified part was covered by these two BACs (Figure 1C), which detected moderate to high-level amplification in eight of the 10 sarcomas analysed (Figure 1B).

The 1q21 amplicon, as represented by YAC 789f2, was present in all tumours with 1q21-q23 amplification analysed here [3,16] (and unpublished results), and it was generally present at higher copy numbers than the amplicon near *APOA2 *in 1q23. This pattern seemed neither to be dependent on the sarcoma subtype, nor on the aggressiveness of the tumour. Both amplicons were for instance observed in WDLS (LS2 and LS21) as well as in the more aggressive leiomyosarcomas. However, the results presented here indicate that target genes located in 1q23 may be important for tumour development or progression in a subset of sarcomas, as this clearly is a separate amplicon.

Amplicon mapping was confirmed by array CGH using tiling-path probes for this region (Figure 2). For the most amplified subregion identified by FISH, four of the tumours showed a variable amplification pattern, whereas the other six tumours showed a relative copy number between 1 and 3. The approximately 800 kb region covered by the BACs RP11-5K23 through RP11-384L19 appeared to be the core of the amplicon, where LS6, LS21 and especially MS8x showed high amplification levels. But for LS21 and LS43, the region covered by the BACs RP11-312J18 through RP11-5K23 also showed high levels, suggesting that additional genes may be important for these two cases.

The results obtained by FISH and array CGH were in general consistent, but differences were also observed, in particular for tumours LS3x and LS43. FISH detected high copy numbers in a substantial fraction of the cells in LS3x, whereas array CGH detected a relative copy number around 3. For LS43 it was the other way around, with higher copy numbers detected by array CGH than FISH. Different pieces of the tumour were used to make interphase nuclei for FISH and isolate DNA for array CGH, and the observed discrepancy could thus be explained by tumour heterogeneity. Heterogeneous amplification status has been detected previously for some tumours, like LS21 [16]. Also, normal cells in the tumours would cause amplification levels of the cancer cells to be underestimated by array CGH, and a subpopulation of cells with high amplification levels may go undetected. FISH analysis, although very laborious, would however detect such a subpopulation.

In order to identify possible candidate target genes we used Ensembl and the UCSC Genome Browser, and Southern blot analysis was performed to determine the copy numbers of nine of the candidate target genes (Figure 3). For tumours LS3x, LS21, LS43 and MS8x, the genes *ATF6 *and *DUSP12 *showed the highest amplification level in general, suggesting that one of them may be the target for this amplification. Both genes were located within the two BACs that detected the highest level of amplification.

Dual specificity phosphatase 12 (*DUSP12*) is a member of the VH1-like dual specificity subfamily of protein phosphatases, which may dephosphorylate both phosphoserine/threonine and phosphotyrosine residues [21]. This gene is also termed *YVH1*, being the *Homo sapiens *ortholog of the *Saccharomyces cerevisiae *gene *YVH1 *protein-tyrosine phosphatase. Little is known about the human DUSP12/YVH1 protein function, but *S*. *cerevisiae *YVH1 is involved in cell growth, meiosis and sporulation [22], and inactivation of the *S*. *cerevisiae YVH1 *gene results in a striking increase in yeast doubling time [23]. A similar effect has been observed in the opportunistic fungal pathogen *Candida albicans*, where *YVH1 *also contributes to pathogenicity [24]. Interestingly, the human *YVH1 *gene has been shown to rescue the slow growth defect in yeast caused by disruption of the *S*. *cerevisiae YVH1 *gene [25]. Since *DUSP12 *may be involved in cell proliferation, it is possible that amplification of *DUSP12 *stimulates tumour growth.

In addition, *S*. *cerevisiae *YVH1 has been shown to interact with the yeast pescadillo homolog (YPH1) [26], and this interaction has also been observed in the malaria parasite *Plasmodium falciparum *with the orthologs of YVH1 and pescadillo [27]. Pescadillo is essential for ribosome biogenesis, nucleogenesis and mammalian cell proliferation [28,29], and disruption to its function results in cell cycle arrest [29]. An increased expression of pescadillo protein has been demonstrated in malignant cells [28], implying that pescadillo may contribute toward tumour progression. Thus, the interaction between YVH1 and YPH1 also connects *DUSP12*/*YVH1 *to cell proliferation and cell cycle regulation.

Activating transcription factor 6 (*ATF6*) is a member of the basic-leucine zipper (bZIP) family of transcription factors. It is involved in the endoplasmic reticulum (ER) stress response pathway, and activates expression of genes induced by the ER stress response [30] (and references therein). Interestingly, the ER stress response is already implicated in sarcoma biology, as the *CHOP/GADD153 *gene, involved in these processes [31], is translocated in myxoid liposarcomas [32] and also sometimes amplified in sarcomas [33].

*ATF6 *has been shown to activate the promoters of the genes glucose-regulated protein 78 kDa and -94 kDa (*GRP78 *and *GRP94*) [34,35], which function as ER chaperones. *GRP78 *is also termed immunoglobulin heavy chain binding protein (*BiP*) and heat shock 70kDa protein 5 (*HSPA5*), while *GRP94 *is also termed tumour rejection (gp96) antigen 1 (*TRA1*).

*GRP78 *and *GRP94 *have been shown to protect cells against apoptosis [36,37] (and references therein), and this anti-apoptotic function suggests that induction of these genes could lead to cancer progression and drug resistance in neoplastic cells. Several studies have correlated over-expression of *GRP78 *and *GRP94 *with tumour growth [36,37] (and references therein), most likely because of inhibition of apoptosis. Thus, it is possible that amplification of *ATF6 *could lead to over-expression of *GRP78 *and *GRP94*, thereby giving the cells a growth advantage or resistance to chemotherapy.

The expression levels of *ATF6 *and *DUSP12 *were determined by quantitative real-time RT-PCR (Figure 4), and in general the expression level of *ATF6 *and *DUSP12 *reflected the amplification level in the different tumours. LS21 and MS8x showed the highest level of expression, with *DUSP12 *being as high as 25-fold over-expressed in MS8x. *DUSP12 *was expressed significantly higher than *ATF6 *in these two tumours (p < 0,001 for MS8x and p < 0,01 for LS21), and also in LMS2x and MFH19 (both p < 0,001). Thus, except for LS3x, *DUSP12 *is significantly higher expressed than *ATF6 *in all the tumours that showed at least 2-fold over-expression of either or both genes, suggesting that *DUSP12 *may be the real target for the amplification.

In order to investigate whether amplified *ATF6 *is active, the expression levels of *GRP78 *and *GRP94 *were determined (Figure 4). Interestingly, both genes were also over-expressed in the tumours that showed high expression of *ATF6 *(LS3x, LS21 and MS8x). *GRP78 *showed especially high levels, being over-expressed 8–9 fold in LS21 and MS8x. In addition, expression of both genes was increased 2-fold in MFH19 and also in LMS2x for *GRP78*, two tumours with similar expression level of *ATF6 *as the tumours with normal copy number of *ATF6*. However, the expression level of *GRP78 *and *GRP94 *in these two tumours is lower than what is observed in the tumours with over-expression of *ATF6*, and may possibly be caused by other regulatory mechanisms. Thus, amplification and over-expression of *ATF6 *seem to cause over-expression of particular *GRP78 *but also *GRP94*, thereby implying that amplification of *ATF6 *has a functional role.

## Conclusion

Based on their consistent association with this amplicon and possible roles in promoting cell growth, both *ATF6 *and *DUSP12 *represent interesting candidate target genes for the 1q23 amplification in sarcomas. *DUSP12 *seems to be the most likely target based on the significantly higher expression in a subset of the tumours, but since relevant genes activated by *ATF6 *also are highly expressed in tumours with over-expression of *ATF6*, both genes are considered potential targets. Further functional analyses are required to determine the role of these proteins in sarcoma development or progression.

## Methods

### Specimens

Ten sarcomas with known alterations of 1q21-q23 were analysed in this study, five liposarcomas (LS), two leiomyosarcomas (LMS), two malignant fibrous histiocytomas (MFH) and one malignant peripheral nerve sheath tumour (MPNST) (previously termed malignant schwannoma, MS). Histopathological and clinical characteristics of these tumours have been previously described [16,17].

### Fluorescence *in situ *hybridisation (FISH)

Fifty-nine overlapping BACs were used as probes for FISH. All the BACs were from the clone library RPCI-11 [38], kindly provided by the Wellcome Trust Sanger Institute . The precise localisation of each BAC was based on sequence alignment by basic local alignment search tool (BLAST), performed by the Wellcome Trust Sanger Institute.

BAC DNA was isolated by standard methods and labeled with biotin-16-dUTP or digoxygenin-11-dUTP (Roche) by use of the BioNick Labeling System (Invitrogen). For each hybridisation, 300 ng of labeled DNA was ethanol precipitated together with 10 μg human Cot-1 DNA (Invitrogen). The precipitated DNA was dissolved in 15 μl hybridisation buffer (50 % formamide, 10 % dextran sulphate, 2 × SSC).

Preparation of interphase nuclei from tumour tissue was done as previously described [16]. The slides were thawed and immersed in 70 % ethanol for at least 1 hour, air-dried and treated with 0,4 mg/ml pepsin for 20 minutes at 37°C. After this, the slides were washed three times for 5 minutes in 1 × PBS, fixated in 1 % formaldehyde/1 × PBS for 10 minutes at room temperature and washed three times for 5 minutes in 1 × PBS. The slides were dehydrated in ethanol (70, 90, 96 and 100 %) and air-dried.

The slides were denatured for 2 minutes at 74°C in 70 % de-ionized formamide/2 × SSC, immediately transferred to ice-cold 70 % ethanol, further dehydrated and air-dried. The labeled DNA was denatured for 10 minutes at 80°C, prehybridised for at least 30 minutes at 37°C and applied to slides at room temperature. Hybridisation was performed overnight at 37°C. After hybridisation, slides were washed three times for 10 minutes in 50 % formamide/2 × SSC at 45°C, and three times for 10 minutes in 0,1 × SSC at 45°C.

Signals from biotin-labelled probes were detected with avidin-Cy3 (Amersham Biosciences), and for detection of digoxygenin-labelled probes we used a FITC-labelled sheep-anti-digoxygenin antibody followed by ALEXA 488-labelled donkey-anti-sheep (Molecular Probes). For probes that gave weak signals, signals were amplified by deposition of FITC- or biotin-labelled thyramides, essentially as described in the protocols supplied by the manufacturer (TSA-direct and -indirect, NEN™ Life Science Products). Signals from biotinylated thyramides were detected with FITC-labelled streptavidin (NEN). The interphase nuclei were counterstained with 4',6-diamino-2-phenylindole (DAPI) and mounted in anti-fade solution (Vector Laboratories).

Hybridised slides were examined visually using a Zeiss Axioplan microscope equipped with appropriate filters for excitation of DAPI, DAPI/FITC or DAPI/Rhodamine (Cy3). The slides were manually scanned at 63 × or 100 × magnification with DAPI excitation to localise the interphases. For each probe, the number of spots was counted in at least 100 nuclei. Amplification levels were grouped into three categories; normal (two signals), moderate (3–9 signals) and high (10 or more signals).

### Microarray-based comparative genomic hybridisation (array CGH)

Genomic microarrays covering the 1q12-q25 region were made using overlapping BACs and P1 artificial chromosomes (PACs). The BACs were from the clone library RPCI-11 [38], whereas the PACs were from the clone libraries RPCI-1, -3, -4 and -5. All clones were kindly provided by the Wellcome Trust Sanger Institute . The precise localisation of each BAC and PAC was based on sequence alignment by BLAST, performed by the Wellcome Trust Sanger Institute.

The results from 15 of the BACs, covering the region in 1q23 that showed highest amplification level by FISH, are presented here. The dataset with these 15 BACs has been submitted to ArrayExpress, the microarray database of the European Bioinformatics Institute (, accession number E-MEXP-427) [20].

Isolation of BAC and PAC DNA, amplification by DOP-PCR and preparation of microarrays were done as previously described [39]. The PCR products were arrayed in quadruplicate onto amine-binding slides (CodeLink, Amersham Biosciences) using a MicroGrid II arrayer (BioRobotics).

Genomic DNA from tumour tissues was isolated by standard methods as described previously [40]. Normal female or male DNA (Promega) was used as a reference. Labeling of the DNA was done as previously described [39], with a few modifications. Here, 500 ng total genomic DNA was labeled using 1,5 μl 1 mM Cy3-dCTP or Cy5-dCTP (Amersham Biosciences) in a total volume of 100 μl.

The labeled tumour and reference DNA were combined and ethanol precipitated together with 135 μg human Cot-1 DNA (Invitrogen). The precipitated DNA was dissolved in 108 μl hybridisation buffer (50 % formamide, 10 % dextran sulphate, 4 % SDS, 2 × SSC) and 4 μl 100 mg/ml yeast tRNA (Invitrogen). The DNA was denatured for 10 minutes at 70°C and prehybridised for at least 30 minutes at 37°C.

Hybridisation was performed using an automated hybridisation station, GeneTAC (Genomic Solutions/Perkin Elmer), agitating the hybridisation solution for 48 hours at 37°C. After hybridisation, the slides were washed with 50 % formamide/2 × SSC at 48°C, 2 × SSC/0,1 % SDS at 48°C and PN-buffer (0,1 M NaH_2_PO_4 _plus 0,1 M Na_2_HPO_4 _to obtain pH 8/0,1 % NP-40) at 25°C. For all three solutions, the hybridisation station washed 5 cycles with a flow time of 20 seconds and a hold time of 40 seconds for each cycle. After removal from the hybridisation station, the slides were rinsed briefly in 0,05 × SSC and dried by spinning in a centrifuge.

The arrays were scanned by use of an Agilent G2565BA scanner (Agilent Technologies). The acquired microarray images were analysed using GenePix Pro 4.1 (Axon Laboratories). The spots were automatically segmented and manually adjusted where necessary. The fluorescent intensities and the local background of the two dyes were calculated for each spot. Further data processing, including filtering and normalisation, was done using M-CGH, a MATLAB toolbox specifically designed for this purpose [41].

### Southern blot analysis

Eleven human cDNA clones were used as probes for Southern blot analysis. The clones for the nine candidate target genes were from the I.M.A.G.E. Consortium [LLNL] cDNA clones library [42], provided by Research Genetics. The I.M.A.G.E. Consortium CloneID and GenBank accession numbers of the clones are as follows: *ATF6 *(417251, W87752); *CAPON *(1860405, AI198232); *DUSP12 *(843328, AA485951); *FCGR2A *(868380, AA634109); *FCGR2B *(138369, R68106); *FCGR3A *(450155, AA703460); *FCGR3B *(51447, H20822); *FREB *(291871, W02963) and *HSPA6 *(2310335, AI654494).

In addition, a cDNA for *APOA2 *[43] was used. As a control probe, the apolipoprotein B (*APOB*) gene, located in the p24 region of chromosome 2, was used [44]. Previous work has shown no amplification of this chromosomal region in neither our sarcoma panel [3,4,45] nor liposarcomas from other groups [2,46]. All cDNA clones were sequence verified.

DNA extraction from tumour tissues, digestion, preparation of filter blots and hybridisation were done as previously described [40]. Quantitation of signal intensity was done by two-dimensional densitometry on a Molecular Dynamics laser densitometer. To correct for unequal sample loading, the gene-specific signals were calibrated to the relative signals obtained from the *APOB *control probe. Amplification levels were calculated by comparison with signals from normal controls (leukocytes), and grouped into five categories; <2 (normal), 2–3, 3–5, 5–10 and >10.

### Quantitative real-time reverse transcription PCR (RT-PCR)

Quantitative real-time RT-PCR was performed using TaqMan^® ^Gene Expression Assays (Applied Biosystems). The expression level was determined for the candidate target genes *ATF6 *(assay ID Hs00232586_m1) and *DUSP12 *(assay ID Hs00170898_m1). In addition, the expression level of *GRP78 *(also termed *HSPA5*, assay ID Hs00946088_g1) and *GRP94 *(also termed *TRA1*, assay ID Hs00427665_g1) was analysed. The genes *B2M *(assay ID Hs99999907_m1), *GAPDH *(assay ID Hs99999905_m1) and *TBP *(assay ID Hs99999910_m1) were used as endogenous controls for normalization. These housekeeping genes were chosen since they showed low variability by microarray expression profiling of our panel of sarcomas (Namløs, Berner, Myklebost *et al*., unpublished).

Frozen tumour tissue was pulverized in liquid nitrogen, and total RNA was extracted using Trizol (Invitrogen) according to the manufacturer's instructions. Universal Human Reference RNA (Stratagene) was used as a reference. cDNA synthesis was performed using the High-Capacity cDNA Archive Kit, essentially as described in the protocols supplied by the manufacturer (Applied Biosystems).

The PCR amplification was performed according to the manufacturer's instructions using the ABI Prism 7000 Sequence Detection System (Applied Biosystems). The cycling conditions comprised 10 minutes polymerase activation at 95°C and 40 cycles of 95°C for 15 seconds and 60°C for 1 minute. Each assay included (in triplicate): a standard curve of four serial dilutions of the Universal Human Reference RNA cDNA (ranging from 50 ng to 50 pg), a no-template control and 2 ng of each tumour cDNA.

Gene expression in the tumours was determined from the standard curves, and the expression level of *ATF6*, *DUSP12*, *GRP78 *and *GRP94 *was normalized with the average expression of the three endogenous controls.

## Authors' contributions

SHK performed the experiments and analyses and drafted the manuscript. JMB participated in performing the Southern blot experiments and analyses. LAMZ participated in performing the array CGH experiments and analyses. SGG provided genomic clones and information. WLK and JWG participated in establishing the array CGH technology. AF and OM conceived of the study, participated in its design and coordination and helped to draft the manuscript.
